# Physical function and psychosocial outcomes after a 6-month self-paced aquatic exercise program for individuals with myalgic encephalomyelitis/chronic fatigue syndrome

**DOI:** 10.1007/s00421-025-05759-5

**Published:** 2025-04-05

**Authors:** Suzanne Broadbent, Sonja Coetzee, Angela Calder, Rosalind Beavers

**Affiliations:** 1 School of Health, University of the Sunshine Coast, PO Box 5280, Sunshine Coast MC, QLD 4560 Australia; 2 Faculty of Health, Southern Cross University, PO Box157, Lismore, NSW 2480 Australia

**Keywords:** ME/CFS, Aquatic exercise, Fibromyalgia, Physical capacity, Fatigue, Depression

## Abstract

**Purpose:**

A randomized-controlled trial to investigate the efficacy of a 6-month self-paced aquatic exercise intervention on physical function, symptoms and psychosocial measures in individuals with myalgic encephalomyelitis/chronic fatigue syndrome (ME/CFS).

**Methods:**

Thirty-two individuals diagnosed with ME/CFS (55.0 ± 13.9 yr) were randomized into an intervention group (INT, *n* = 17) or control group (CON, *n* = 15) for a 6-month trial of two 20-min sessions per week of self-paced aquatic movements and stretches. Pre- and post-intervention outcomes included physiological measures, 6-min walk test, hand-grip strength, Sit-to-Stand, Apley’s shoulder test, Sit–Reach test, perceived exertion, fatigue (FACIT), anxiety/depression (HADS) questionnaires, and tiredness and pain scores (VAS 0–10 scale).

**Results:**

The INT group significantly increased walk test distance (13.7%, *P* < 0.001), Sit-to-Stand scores (33.7%, *P* < 0.001) and peak expiratory pulmonary flow (12.9%, *P* = 0.028) post-intervention. Fatigue (29.5%, *P* = 0.005), depression (21.7%, *P* = 0.010), combined anxiety/depression scores (16.9%, *P* = 0.047) and resting diastolic blood pressure (4.8%, *P* < 0.001) also significantly improved for the INT group. Sit–Reach scores were significantly lower for the INT group compared to CON post-intervention (− 4.0 ± 10.4 vs + 4.3 ± 10.7 cm, *P* = 0.034). There were no adverse events or worsening of symptoms during the trial.

**Conclusions:**

Self-paced, low–moderate-intensity aquatic exercise improved walk distance, lower limb strength, fatigue, depression and peak expiratory flow without worsening ME/CFS symptoms. This mode of low-intensity physical activity may confer mental health and physical benefits provided the activity is self-paced and within patient energy limits.

**Trial registration number:**

Australian and New Zealand Clinical Trials Registry ANZCTRN12618001683224.

**Supplementary Information:**

The online version contains supplementary material available at 10.1007/s00421-025-05759-5.

## Background

Myalgic encephalomyelitis/chronic fatigue syndrome (ME/CFS) is a complex, debilitating multisystem medical condition where the primary symptoms are post-exertional malaise (PEM), fatigue, cognitive dysfunction and unrefreshing sleep for more than 6 months (McManimen and Jason 2017; Lim and Son 2020; Vollestad and Mengshoel 2023). Neurological, immunological, autonomic and gastrointestinal symptoms may be present (Lim and Son 2020; Vollestad and Mengshoel 2023), and there is also some evidence of underlying mitochondrial dysfunction which would contribute to PEM (Holden et al. 2020; Huth et al. 2020).

Recommendations for physical activity for ME/CFS have been fraught with controversy in previous years. Early studies (White et al. 2011) and systematic reviews (Van Cauwenbergh et al. 2012; Larun et al. 2017) that reported “graded exercise therapy” (GET), largely consisting of aerobic exercise of up to 30 min duration with fixed incremental progressions, as safe and beneficial for ME/CFS, are no longer supported. Current National Institute for Health and Care Excellence and Mayo Clinic guidelines (NICE 2021; Bateman et al. 2021) outline best-practice recommendations for physical activity (PA) which must be individualized; undertaken within a ME/CFS management program and overseen by a trained exercise therapist; within each person’s energy limits; and flexible, allowing for regression and progression only if this is within energy limits and does not worsen the patient’s symptoms.

Individuals with ME/CFS experience a wide range of symptoms and there is considerable variance in the severity of the condition. Approximately a third of patients have reported improvements in their health over time, but the illness remains persistent in many adult patients even after specialist treatment (Collin and Crawley 2017). Appropriate modes of PA are uncertain (Galeoto et al. 2018), given the risk of symptom exacerbation, yet remaining sedentary may also increase the risk of developing conditions, such as type 2 diabetes, cardiovascular disease and cancer (NICE 2021). ME/CFS is also compounded by the high percentage of patients who suffer from concurrent fibromyalgia syndrome (FMS), which includes muscular pain in specific areas, pain amplification and allodynia (Zamuner et al. 2019; Sarzi-Puttini et al. 2021; Siracusa et al. 2021).

Discounting previous controversial GET studies, there are limited literatures for the efficacy of exercise and physical activity for ME/CFS. Wallman et al. (2004) reported that 12 weeks of paced aerobic exercise in short sessions (walking, swimming, cycling) every second day improved aerobic capacity, depression and mental fatigue without worsening symptoms. Short intermittent and continuous blocks of low-intensity, self-paced indoor cycling, with regression if needed, did not cause PEM or increase symptoms (Broadbent and Coutts 2016). Gordon and colleagues (2009; 2010) conducted successful short-term strength training studies with adolescent ME/CFS patients. Other studies with positive findings include a 5-week aquatic exercise pilot study (Broadbent et al. 2018) and alternative forms of physical activity, such as tai chi (Wu et al. 2022), yoga and qui gong (Fricke-Comellas et al. 2024). There is more evidence for therapeutic benefits of low-to-moderate-intensity physical activity for FMS, including aquatic exercise, tai chi, stretching and resistance training (Gusi et al. 2006; Busch et al. 2011; Sosa-Reiner et al. 2017; Andrade et al. 2019; Zamunar et al. 2019; Fail et al. 2021; Bekaryssova et al. 2025). However, we still lack guidelines for implementing appropriate physical activity for both ME/CFS and FMS patients.

We have previously reported benefits of self-paced, low-intensity aquatic exercise for ME/CFS patients in a short-term pilot study (Broadbent et al. 2018), but a longer duration, controlled feasibility trial was necessary to determine benefits and sustainability for rehabilitation and clinical practice. Aquatic rehabilitation and hydrotherapy are evidence-based modes of management for a wide range of musculoskeletal and neurological conditions (Bidonde et al. 2014; Busch et al. 2011; Fail et al. 2021). The hydrodynamic properties of water (buoyancy, viscosity, hydrostatic pressure) are well-documented for enhancing muscle relaxation, strength, cardiovascular function, blood circulation and autonomic function, and for reducing pain, fatigue and edema (Becker 2009; Fail et al. 2021). Furthermore, this mode of PA is well-tolerated and usually enjoyed by participants, with high compliance (Broadbent et al. 2020). However, the majority of clinical population studies have been short term, with chronic disease benefits shown between 4 and 26 weeks, (Fail et al. 2021), so for relevancy to clinical practice and participant adherence, it was important to investigate aquatic intervention effects with a longer-term study.

The aim of this 6-month RCT was to investigate the potential physical and psychosocial benefits from a longer aquatic exercise program, with regard to establishing the effectiveness, feasibility and safety of self-paced ME/CFS rehabilitation for clinical practice.

## Methods

### Design

This randomized controlled trial (RCT) was conducted over 6 months at two locations and approved by the Human Research Ethics Committees of both Southern Cross University, NSW (ECN-18–131) and University of the Sunshine Coast, QLD (A181115). The trial was registered with Australian and New Zealand Clinical Trials Registry ANZCTRN12618001683224.

### Participants

Thirty-four individuals diagnosed with ME/CFS were initially recruited from the Sunshine Coast region (QLD) and from the Northern Rivers area (NSW) through university websites, flyers, ME/CFS support groups and local media advertising. Participants at each study site were randomized into an intervention group (INT) or usual care/no exercise (CON) using a computer random number generator. A required sample size of 34 was estimated using a G*Power (V3.1.9.7) analysis to achieve a power of 80%. With a drop-out of two participants from the CON group, the final cohort size of 32 was considered still sufficient to detect a medium effect size of 0.5 and significant differences with *P* < 0.050. The final cohort included seven CON and seven INT participants at the University of the Sunshine Coast site and eight CON and ten INT participants at the Southern Cross University.

Inclusion criteria were a medical diagnosis of ME/CFS according to the Canadian Consensus Criteria (or the Fukuda Criteria if that was standard at the time of diagnosis); aged from 18 to 80 years; not participating in regular vigorous exercise or physical activity; able to communicate in English; able to give informed signed consent; and able to commit to the time required for the study. Exclusion criteria included cardiovascular, metabolic, renal, endocrine, autoimmune, neurological and/or inflammatory conditions that made physical activity hazardous; severe chronic obstructive pulmonary disease or uncontrolled asthma; severe ME/CFS with extreme symptoms that incapacitated the individual; any other diagnosed medical condition causing chronic or severe fatigue (e.g., multiple sclerosis); current musculoskeletal injury preventing physical activity; infectious disease; non-swimmer; allergic to chlorine or other pool chemicals; pregnancy.

### Procedures

All participants had initial discussions with the research teams and gave written informed consent. Participants attended the exercise science research laboratories at Southern Cross University or University of the Sunshine Coast for pre-intervention data collection, which included medical history and demographic data (Table 1 and Supplementary Table). All participants completed a health screening including the Adult Pre-exercise Screening Tool V2 (ESSA 2019), height, weight, BMI, resting heart rate (HR), blood pressure and oxygen saturation (O2sat%); orthostatic blood pressure test; lung function; and physical function tests (6-Minute Walk Test (6MWT); 60 s Sit-to-Stand; bilateral hand grip; bilateral Apley’s shoulder test). Rate of perceived exertion (RPE) (Borg 1982), heart rate and O2sat% were recorded every 2 min during the walk test, and participants were asked to record their tiredness and pain (if any) immediately and 24-h post-6MWT using 0–10 Visual Analog Scales (VAS). Psychosocial questionnaires included the Functional Assessment of Chronic Illness Therapy – Fatigue (FACIT) (Cella et al. 2019); Hospital Anxiety and Depression Scale (HADS) (Zigmond and Snaith 1983); and quality of life (SF-36) (Ware 1999). In addition, all participants underwent a full blood test with white cell differential, conducted at a qualified pathology center, within 2 days of their pre-intervention screening. All outcome measures were reassessed post-intervention.

**Table 1:** Participant contributing factors for ME/CFS and co-morbidities (*n* = 32)

Factors	Frequency INT group (*n* = 17)	Frequency CON group (*n* = 15)	Percentage of total cohort %
ME/CFS cause or contributing factor(s)			
Viral infection(s)	16	9	78.1
Bacterial infection	2	2	12.5
Stress	4	2	18.8
Tick bite	0	1	3.1
Shingles	1	0	3.1
Insecticide exposure	1	1	6.2
Comorbidities/concomitant conditions			
Cardiovascular disease	5	4	28.1
Fibromyalgia	6	5	34.4
POTS	3	3	18.6
Autoimmune disorder	6	6	37.5
Hypertension	2	2	12.5
Hypotension/orthostatic intolerance	4	9	40.6
Pulmonary disease (including asthma)	7	2	28.1
Hyperglycemia	5	3	25.0
Hyperlipidemia	6	4	31.3
Neurological	3	3	18.6
Clinical Depression	6	7	40.6
Anxiety disorder	5	7	37.5
Other diagnosed mental health disorder	4	4	25.0
Migraines	9	10	59.4
Anemia	4	6	31.3
Cancer (previous)	3	2	15.6
Gastrointestinal	2	2	12.5
Endocrine	5	6	34.4
Renal disorder	0	1	3.1
Gynecological	0	1	3.1
Osteoarthritis	7	5	37.5
Osteoporosis	3	4	21.9
Lower back pain (intermittent)	3	1	12.5
Allergies	5	6	34.4
Sleep Disorder	12	11	71.9

*POTS* Postural Orthostatic Tachycardia Syndrome; *MH* mental health; *BP* blood pressure

The aquatic exercise intervention had been piloted in 2016 as a 5-week intervention (Broadbent et al. 2018) and the typical structure of the sessions is described there. The current RCT followed the same intervention structure for 6 months, with the addition of the HADS and SF-36 questionnaires pre- and post-intervention. The settings for each intervention were indoor, climate-controlled salt-water chlorinated pools at Southern Cross University and Flinders Academy (Sunshine Coast). Pools at both locations had a temperature of 27–29^O^ C and water depth varied from 1.2 to 1.8 m. The supervised exercise sessions were twice weekly lasting approximately from 15 to 20 min, and there was 48–72 h recovery between each session. Participants were gradually able to increase the duration of their session during the study, if symptoms and energy allowed. Participants were constantly reminded to remain within their energy ‘envelopes’ and to reduce session time or remain home if they felt tired or unwell. There were supervisors at every session; one supervisor led the session in the water and/or from the pool deck while the others monitored the clients and collected data. Heart rates were monitored and recorded before, at 10-min intervals and after each session, using either a Polar T31 HR monitor (Oulu Finland) at Southern Cross pool or Garmin Vivosmart 4 fitness tracker (Garmin Australia) wrist-worn monitor at the Sunshine Coast pool. RPE was also recorded at 10-min intervals during each session. Buoyancy belts (HART Sport, Australia) were provided to participants if they wished, and the majority of movements were performed in chest-deep water. Access to a ramp or steps, and deep-water platforms was provided at either location. Pool noodles, kickboards and buoyancy belts were available for specific movements during sessions.

Exercise sessions included 5 min of a gentle warm-up and 5-min cool-down, which included stretching. All exercises were self-paced with participants encouraged to move within their energy limits. Low-intensity water-walking was followed by a short selection of upper and lower body exercises through different ranges (e.g., squats, calf raises, lunges, tai chi range of motion movements, pool noodle arm and trunk exercises, arm circling, wall push-ups), which were varied weekly. Pool noodle “cycling”, breast or side-stroke swimming, water-walking and “skiing” were also included as short forms of low-intensity aerobic activity. Participants completed repetitions of movements at their own pace in a set period of time e.g., self-selected calf raises that could be done in 30 s. Simple floating was used as a relaxation exercise if participants felt tired. Stretching of the main muscle groups was completed after the cool-down. The session supervisors were trained clinical exercise physiologists, assisted by final year trained clinical exercise physiology students, with the lead staff at both locations experienced in delivering aquatic exercise rehabilitation to a variety of different chronic condition clients. Participants in both INT and CON groups were asked to complete daily diaries for the duration of the study with the aim of recording fatigue and other symptoms, energy levels, sleep and other factors which might affect their health. These were to be collected post-intervention.

Within one-week post-intervention, physical and psychosocial assessments were completed at the exercise science laboratories and blood tests at the pathology centers. Diaries were collected and participants completed a study exit survey with the researchers. This qualitative data will be published separately.

### Statistical analyses

Data are expressed as percentages and fractions of cohort numbers or mean ± standard deviation, percent changes and a modified Cohen’s d effect size (ES) with magnitudes interpreted as trivial (0.0–0.2); small (0.2–0.6); moderate (0.6–1.2); large (1.2–2.0); very large (> 2.0) (Batterham and Hopkins 2006). Quantitative data were analyzed using a general linear model with repeated measures (SPSS V29, IBM Corporation NY) with 2-group between-subject (Timepoints) and within-subject (Group) comparisons and an a priori significance level of *P* < 0.05. Intention-to-treat was used to impute missing quantitative data.

## Results

### Participant characteristics

The participant mean age was 55.0 ± 13.9 yr. The average time since symptom onset (TSSO) was 17.1 ± 11.0 yr and time since diagnosis (TSD) was 14.4 ± 10.5 yr. The most common contributing factors to the development of ME/CFS were viral infections (Table 1) and multiple co-conditions were common in all participants. Nearly 75% of total participants were female; the gender composition of the INT group was 12 females and 5 males, and the CON group 12 females and 3 males. Regarding physical activity (PA) levels prior to study entry, 31.25% of the cohort were doing either no PA or only essential daily activities; 59.4% were able to manage short walks; 18.6% did some gardening; 12.5% engaged in yoga and one person did a weekly Pilates session. Nearly 66% of the cohort had never smoked, with 34.4% being previous smokers. Medications, vitamins and supplements taken by participants are shown in the Supplementary Table.

### Physical outcome measures

Physical characteristics data are shown in Table 2 and the pre–post changes in physical assessments are shown in Table 3. Individual participant pre-to-post data is also shown in Supplementary Figs. 1–8. The INT group significantly decreased resting diastolic blood pressure (*P* < 0.001, ES 0.9) and showed post-intervention increases for lung peak expiratory flow (*P* = 0.028, ES 0.4) (Table 2). The orthostatic hypotension test (supine resting HR and BP then at 1 min and 3 min standing) showed no significant differences in HR and BP between groups at any time point, pre- or post-intervention; there were no changes in either variable pre-to-post for either group.

**Table 2:** Changes in physical characteristics for INT (*n* = 17) and CON (*n* = 15) pre- to post-intervention and between groups at each timepoint

Outcome measure	INT Pre mean ± SD	INT Post mean ± SD	INT Pre – post P value	Pre – post % Change & ES	CON Pre mean ± SD	CON Post mean ± SD	CON Pre – Post P value	Pre – post % Change & ES	Pre- between group P value	Post-between group P value
Height (m)	1.7 ± 0.1	–	–	–	1.7 ± 0.1	–	–	–	–	–
Weight (kg)	77.4 ± 18.9	78.4 ± 18.2	0.116	0.1 0.05	75.2 ± 18.0	75.8 ± 17.8	0.391	0.1 0.03	0.738	0.682
BMI	27.7 ± 6.1	28.1 ± 5.7	0.104	0.1 0.07	26.8 ± 5.7	27.0 ± 5.5	0.397	0.7 0.04	0.675	0.600
Resting HR (bpm)	73.9 ± 12.1	72.9 ± 11.9	0.719	− 1.4 0.08	75.1 ± 12.6	74.2 ± 11.9	0.753	− 1.2 0.07	0.787	0.762
Resting SBP (mmHg)	129.2 ± 19.7	125.4 ± 16.0	0.309	− 2.9 0.2	124.7 ± 21.0	124.5 ± 24.7	0.960	− 0.2 0.01	0.536	0.904
Resting DBP (mmHg)	79.4 ± 9.1	71.1 ± 10.5	< 0.001*	− 4.8 0.9	74.9 ± 9.4	73.2 ± 11.9	0.496	− 2.3 0.1	0.175	0.603
Resting O2sat (%)	97.3 ± 1.2	97.1 ± 2.0	0.552	− 0.2 0.1	97.8 ± 1.1	98.1 ± 1.1	0.527	0.3 0.3	0.214	0.099
FVC (L)	3.5 ± 0.8	3.5 ± 0.8	0.207	0 0	3.6 ± 0.9	3.5 ± 0.9	0.088	− 2.7 0.1	0.730	0.789
FEV1 (L)	2.8 ± 0.7	2.8 ± 0.7	0.914	0 0	2.9 ± 0.8	2.8 ± 0.8	0.089	− 3.4 0.1	0.669	0.883
FVC/FEV1	77.8 ± 5.2	79.4 ± 6.5	0.263	2.1 0.3	78.3 ± 6.3	78.0 ± 6.6	0.807	− 0.004 0.06	0.821	0.560
PEF (L/s)	6.2 ± 2.0	7.0 ± 1.9	0.028*	12.9 0.4	6.6 ± 1.5	6.1 ± 1.6	0.138	− 7.6 0.3	0.551	0.166
FEF25–75%	2.6 ± 0.9	2.7 ± 1.0	0.627	3.8 0.1	2.7 ± 1.0	2.6 ± 1.0	0.460	− 3.7 0.1	0.669	0.971

^*^Significant difference from Pre- to Post-intervention for INT group

**Table 3:** Changes in physical function outcome measures for INT (*n* = 17) and CON (*n* = 15) pre- to post-intervention and between groups at each timepoint

Outcome measure	INT Pre mean ± SD	INT Post mean ± SD	INT Pre – post *P* value	Pre – post % change & ES	CON Pre mean ± SD	CON Post mean ± SD	CON pre – post *P* value	Pre – post % change & ES	Pre- Between Group *P* value	Post-Between Group *P* value
6 MWT distance (m)	391.9 ± 105.5	445.7 ± 89.5	< 0.001*	13.7 0.6	398.2 ± 115.4	417.8 ± 87.7	0.185	4.9 0.2	0.871	0.381
Post-6MWT tiredness (VAS 0–10)	3.3 ± 2.2	3.4 ± 2.5	0.858	3.0 0.04	4.3 ± 2.5	4.6 ± 2.3	0.790	7.0 0.1	0.237	0.200
24-h post- 6MWT tiredness (VAS 0–10)	3.4 ± 2.3	2.8 ± 2.8	0.464	17.6 0.2	3.5 ± 3.3	4.3 ± 2.8	0.455	22.9 0.3	0.976	0.134
Post-6MWT pain (VAS 0–10)	1.7 ± 1.9	1.7 ± 1.7	0.899	0.0 0.0	1.9 ± 2.3	1.8 ± 2.1	0.0.857	5.3 0.05	0.783	0.832
24-h post- 6MWT pain (VAS 0–10)	2.7 ± 2.8	2.3 ± 2.8	0.649	14.8 0.1	2.1 ± 2.5	2.2 ± 2.4	0.890	4.8 0.04	0.515	0.945
Right-hand grip (kg)	27.2 ± 8.9	29.2 ± 7.8	0.153	7.4 0.2	29.8 ± 10.9	27.4 ± 13.2	0.104	− 8.1 0.2	0.476	0.634
Left-hand grip (kg)	23.9 ± 9.8	25.9 ± 9.0	0.152	8.4 0.2	27.1 ± 13.4	25.4 ± 14.1	0.283	− 6.3 0.1	0.442	0.906
Sit-to-stand 60 s (reps)	17.2 ± 7.6	23.0 ± 7.5	< 0.001*	33.7 0.8	21.4 ± 10.3	21.5 ± 8.9	0.911	0.5 0.01	0.193	0.618
Sit–reach test (cm)	− 7.9 ± 12.5	− 4.0 ± 10.4	0.152	49.4 0.3	− 1.0 ± 11.5	+ 4.3 ± 10.7	0.071	430 0.4	0.112	0.034^x^
Right Apley’s Shoulder test (cm)	− 3.6 ± 9.2	− 2.7 ± 8.3	0.338	25 0.1	− 3.7 ± 10.7	− 4.8 ± 9.6	0.324	− 29.7 0.1	0.966	0.521
Left Apley’s Shoulder test (cm)	− 5.6 ± 9.0	− 4.9 ± 9.2	0.770	12.5 0.08	− 2.5 ± 11.5	− 4.8 ± 13.2	0.361	− 92.0 0.2	0.390	0.964

*6MWT* 6-min walk test

^*^Significant difference from Pre- to Post-intervention for INT group

^X^Significant difference between INT and CON groups either Pre- or Post-intervention

The INT group recorded a significantly greater post-intervention 6MWT distance (*P* < 0.001, ES 0.6) compared to baseline, and improved Sit-to-Stand scores (*P* < 0.001, ES 0.8) (Table 3). There were no significant within-group or between-group differences in post-6MWT and 24-h post-6MWT tiredness or pain for either group, pre- and post-intervention. The CON demonstrated greater Sit-Reach scores (*P* = 0.034) compared to the INT.

### Hematology

Hematology analyses found no significant differences between groups pre- and post-intervention, nor any within-group pre-to-post changes, for hemoglobin, red cell count, hematocrit, mean cell volume, total leukocytes, neutrophils, lymphocytes, basophils and eosinophils. The INT group had a significant decrease in platelet concentration from pre-to-post (*P* = 0.010 ES 0.2) with no change for the CON group nor differences between groups pre and post. Pre-intervention, the INT group had a significantly lower monocyte concentration than CON (*P* = 0.015), but there were no pre-to-post changes for either group. All hematology components were within the normal reference ranges.

### Psychosocial outcome measures

**Fig. 1:**
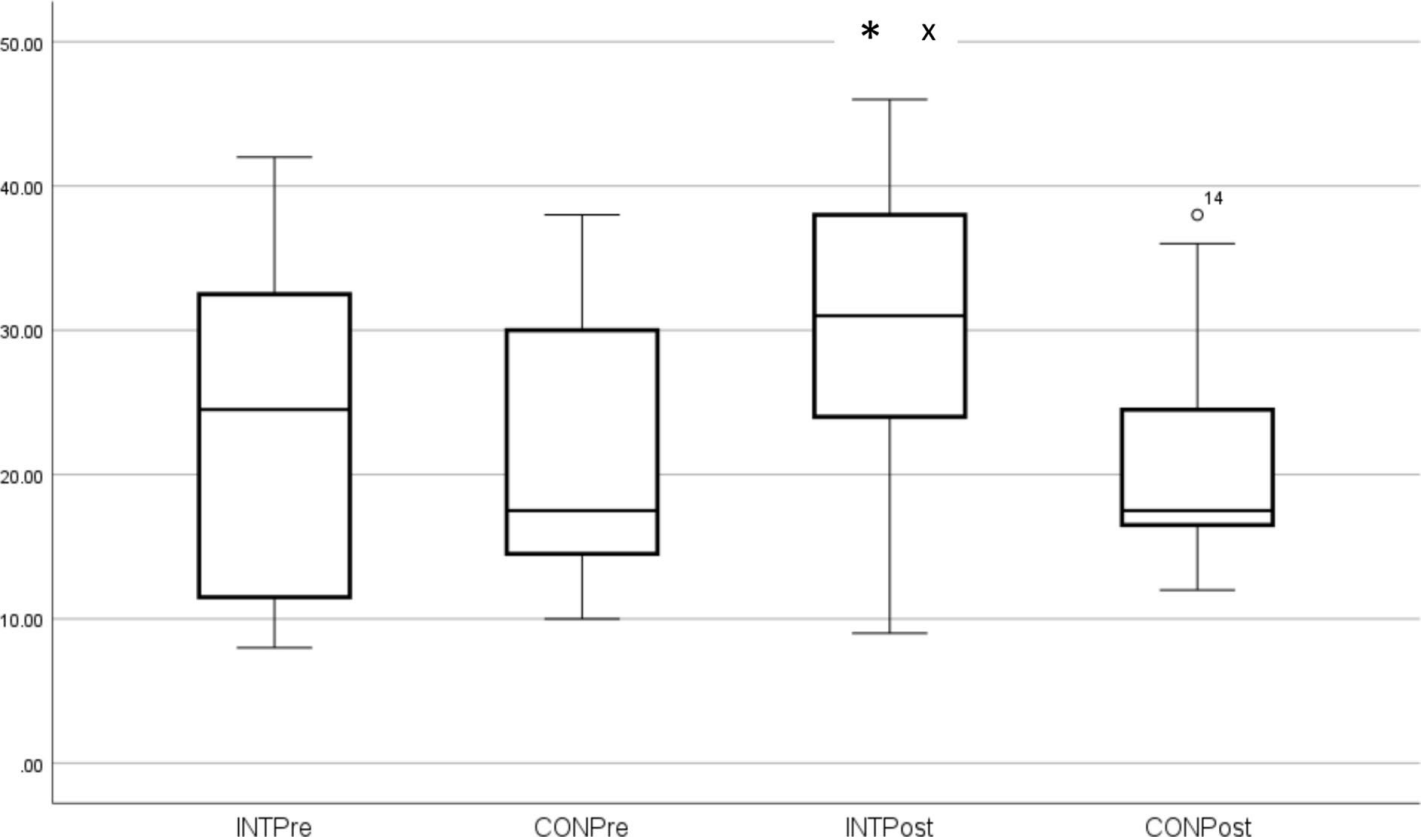
FACIT Scores. *Significant difference pre to post. ^X^Significantly different to CON post-intervention

**Fig. 2:**
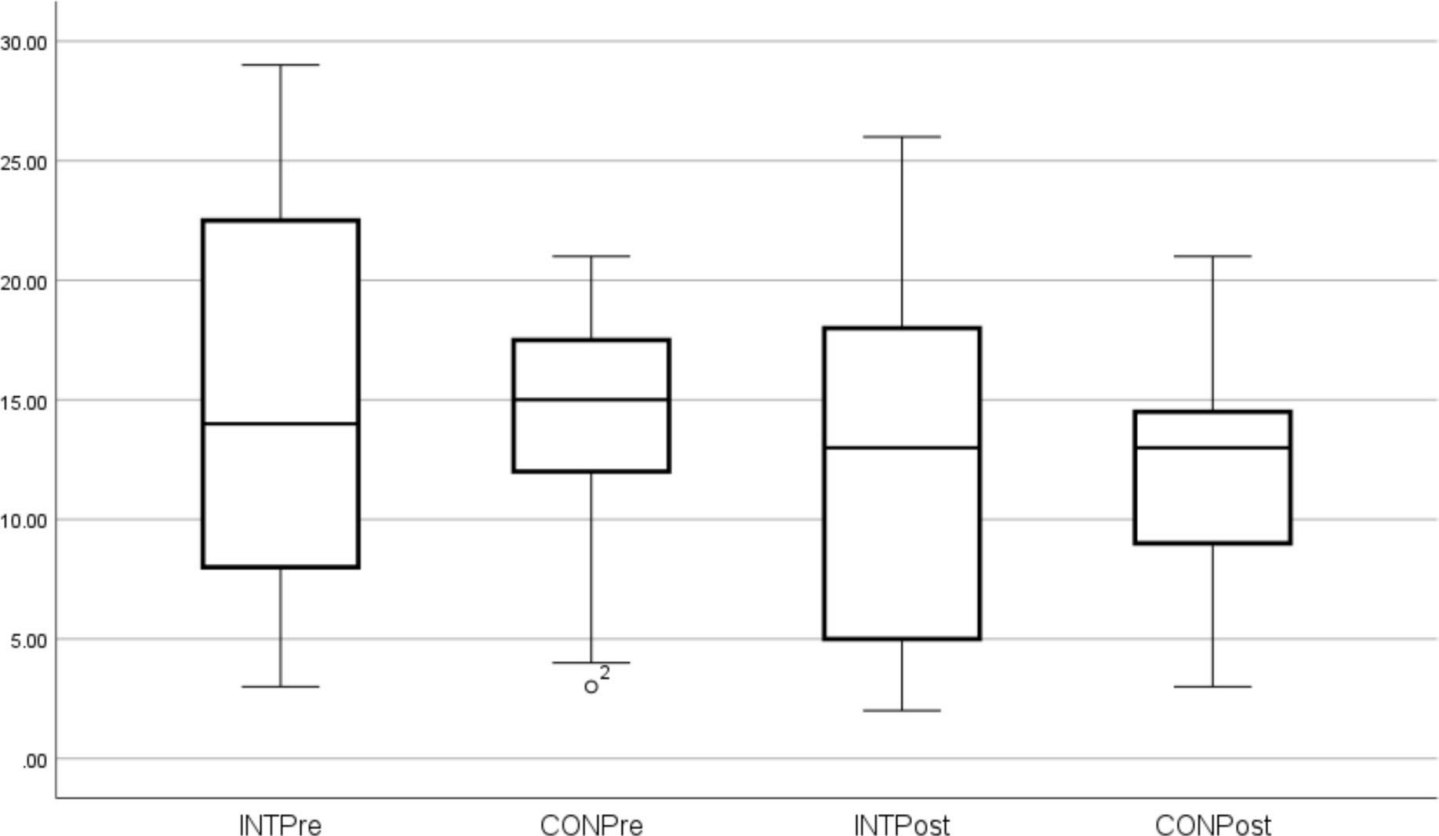
HADS total scores (combined Anxiety and Depression)

**Fig. 3:**
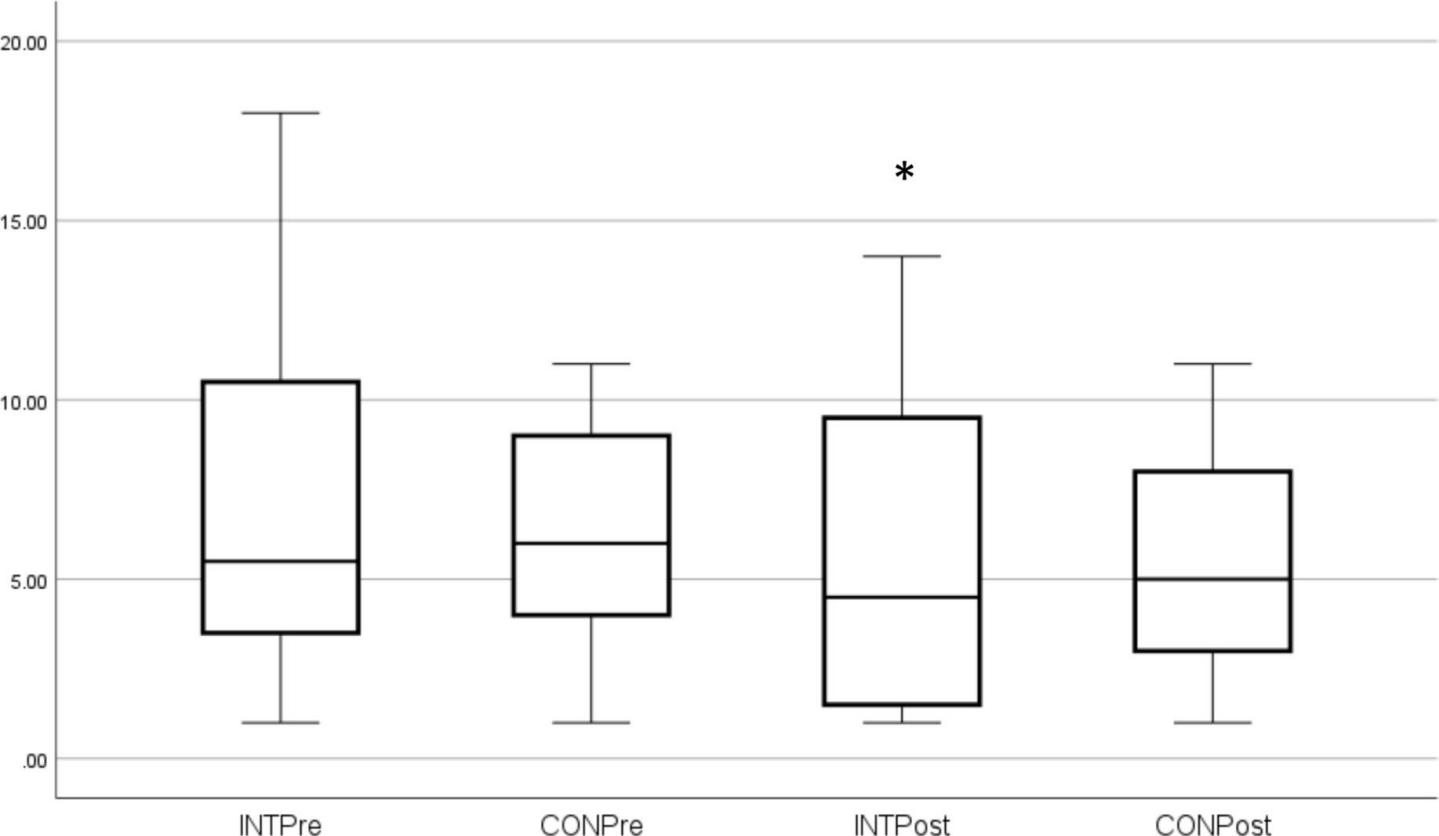
HADS Depression scores. *Significant difference pre to post

**Fig. 4:**
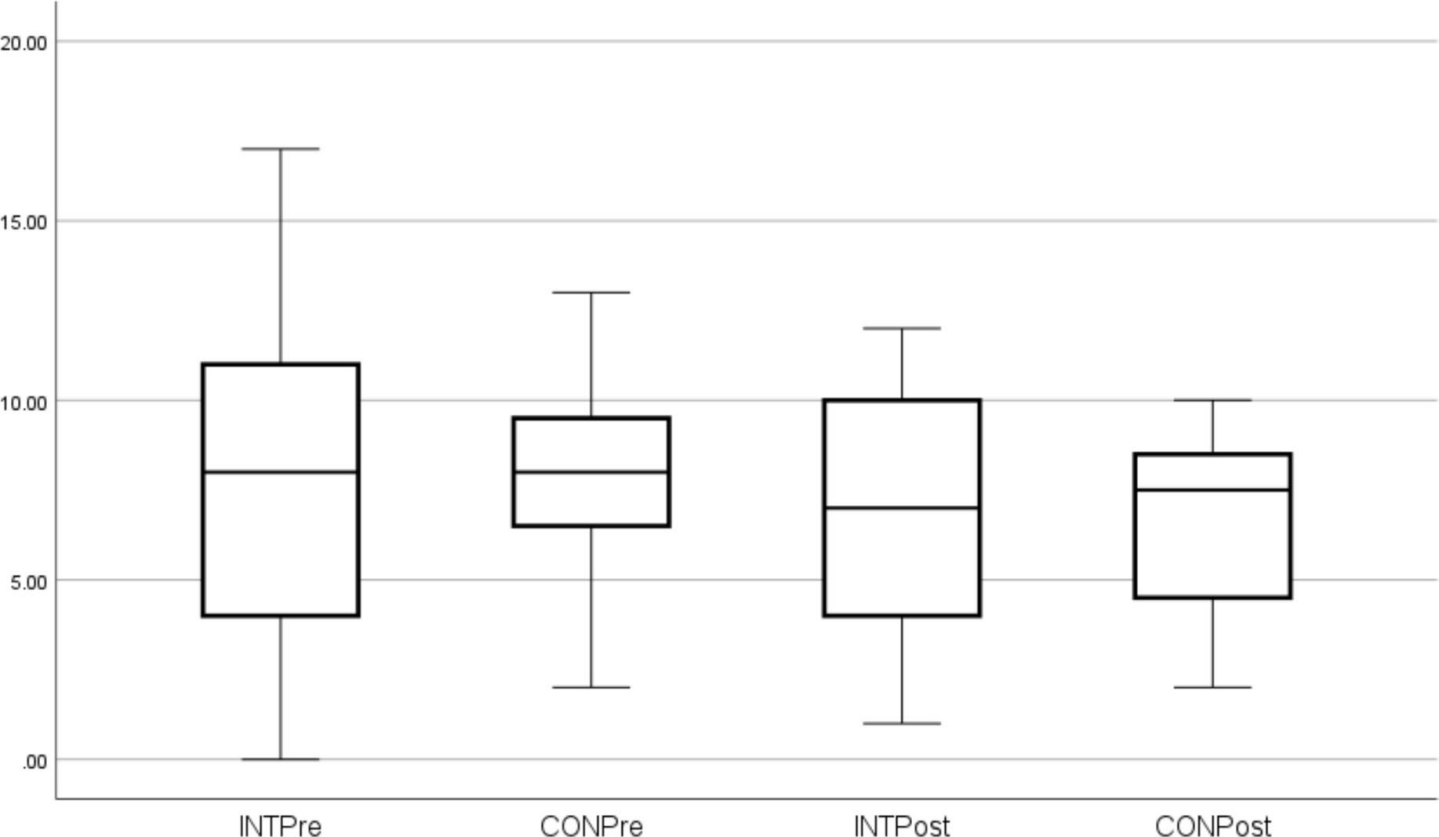
HADS Anxiety scores

These outcome measures (median and SD) are shown in Figs. 1, 2, 3, 4. Individual pre-to-post responses are shown in Supplementary Figs. 9–11. The INT group significantly improved their FACIT-scored fatigue (*P* = 0.005, ES 0.6) pre- to post-intervention and also had reduced fatigue compared to the CON group at that timepoint (*P* = 0.003). The INT group total HADS score (combined depression and anxiety, *P* = 0.047, ES 0.3) and HADS depression (*P* = 0.021, ES 0.3) were significantly reduced in pre- to post-intervention. Of the participants who were taking anti-depressants (40.6% of the cohort), all participants in the INT group reduced their total HADS scores compared to those in the CON group, and all but one INT participant reduced their HADS Depression component score. (Supplementary Figs. 9–11).

### Adherence and safety

There were no reported adverse events or worsening of ME/CFS symptoms during the trial. Intervention attendance was 75% on average for the 6 months.

## Discussion

This RCT investigated physical and psychosocial outcome measures after a 6-month self-paced aquatic exercise rehabilitation program for individuals with ME/CFS, and results confirm many findings from our previous 5-week pilot study (Broadbent et al. 2018). The current study results include increased walk distance/speed and lower limb strength, post-intervention reduced fatigue and importantly, no adverse events or worsening of symptoms during the study. NICE guidelines (2021) state that individually tailored physical activity programs are encouraged where possible but must remain within patient limits. Since there are few recent robust controlled physical activity intervention studies, the impact of different modes of exercise rehabilitation on individuals with ME/CFS is somewhat uncertain. This RCT provides evidence of efficacy and safety for low-intensity aquatic exercise rehabilitation.

The long-term improvements in walk speed/distance and lower limb strength are extremely important in a clinical population who can suffer from PEM and poor physical function, and are due to improved cardiovascular function and muscular adaptations. The benefits of aquatic exercise for increasing cardiovascular capacity in healthy participants and clinical cohorts have been well-documented, including improved cardiac function, systemic and peripheral vascular adaptations and enhanced oxygen consumption (Becker 2009; Meredith-Jones et al. 2011; Bidonde et al. 2014; Zamuner et al. 2019). Our results are supported by previous studies which reported improvements in walk distance after aquatic exercise programs for chronic illness cohorts (Salem et al. 2011; de Souto Araujo et al. 2012; Gianotti et al. 2014; Broadbent et al. 2018). Nearly 60% of our participants were doing short walks (between 5 and 15 min) prior to joining the study, so the significant increase in walk speed and distance with low-intensity aquatic exercise indicates adequate adaptation to physiological stimuli to improve physical capacity (cardiovascular function, peripheral vascular adaptations, oxidative capacity, mitochondrial density and lean muscle mass). Resting diastolic blood pressure also decreased significantly in the INT group and contributing factors are likely to be moderate-intensity exercise-induced cardiovascular adaptations over 6 months, such as lower vascular resistance, greater stroke volumes and increases in dimensions of the ventricles (Becker 2009; Cornelissen and Smart 2013; Torres-Ronda and del Alcatraz 2014: Fukuie et al. 2021). With regard to decreasing diastolic blood pressure, aquatic exercise contributes to the same adaptations as land-based exercise but through hydrostatic pressure effects and the redistribution of blood volumes during repeated immersions (Junior et al. 2020; Angrainie et al. 2023). Thus, there are increases in end-diastolic volume and reductions in systemic and peripheral vascular resistance, which is important for clinical populations given that hypertension is a primary predictor of coronary artery disease (Fail et al. 2021).

There is a paucity of literature reporting outcomes from strength-based physical activity programs for individuals with ME/CFS. Fulcher and White (2000) found that ME/CFS patients had weaker quadriceps muscle strength compared to healthy individuals, which would impact functional capacity. Early studies found strength gains and no adverse effects through group circuit exercise (Karper and Stasik 2003) and a mixed-mode four-week program incorporating light resistance training and hydrotherapy (Gordon and Lubitz 2009; Gordon et al. 2010). Our previous pilot study also found significant gains in hand-grip strength after a 5-week aquatic exercise pilot program (Broadbent et al. 2018). The current trial found no changes in hand grip but a significant improvement in lower limb strength (Sit-to-Stand), which is extremely important for functional mobility, daily activities and for walking. Previous aquatic exercise studies with FMS patients also reported increased knee flexor and extensor strength (Gusi et al. 2006; Tomas-Carus et al. 2009; Bidonde et al. 2014), associated with improved lower limb function. Strength adaptations to specific exercises include changed muscle architecture (e.g., hypertrophy or increased fiber cross-sectional area), enhanced neural activity (e.g., motor unit recruitment and firing) and increased contractile properties (Weakley et al. 2023; Wilson et al. 2023). It is likely that such adaptations were due to including calf raises, squats, lunges, on-the-spot marching, 10 m repeats of water-walking and deep-water lower limb movements. Interestingly, recent research has found that the activation level for lower limb muscles while stepping in water is much lower than that on land, meaning that the exercise will be less demanding than similar land-based movements, and strength gains can occur with a lower level of exertion (So et al. 2023). These exercises produced a greater effect over the longer duration on the RCT compared to the short pilot study, which may reflect the time taken for physiological adaptation of specific lower limb muscle groups (Weakley et al. 2023). Both the cardiovascular and strength adaptations are important for patients to manage functional movements for activities of daily living (ADL) (Gianotti et al. 2014). The lack of change in hand grip strength in the RCT could relate to specificity of hand movements. The intervention did not use a lot of specific hand and forearm strength exercises but rather more general upper and lower body exercises, where water resistance would be a stimulus for increasing muscular strength. However, the results may also reflect the differences in symptom severity between participants, as one study reported correlations between hand grip strength and parameters of ME/CFS severity (Nacul et al. 2018), attributed to greater muscular weakness and fatigue. Another factor may be the difference in sample size between the pilot (*n* = 11) and RCT (*n* = 17) studies, shower greater variance in hand strength in the larger cohorts.

One of the most important findings from this study was that the INT group reported significantly less fatigue (higher FACIT score) post-intervention compared to baseline, similar to findings from our previous pilot study; the INT group also had significantly lower fatigue compared to the CON group post-intervention. This is an exciting result in terms of clinical practice for ME/CFS patients for whom PEM is a primary concern with any type of PA (Vollestad and Mengshoel 2023). Exercise is known to increase blood circulation and oxygen uptake, and to improve mitochondrial density and function, thus providing more energy (Wender et al. 2022). Aquatic exercise may reduce fatigue, both physical and perceived, through buoyancy effects where body weight is unloaded, muscles and joints more relaxed and patients expend less energy (Zamunar et al., 2019). Neuromuscular responses, neural transmissions, and inhibitory mechanisms responsible for feelings of fatigue and nociceptive pain may also be reduced by water immersion (Becker 2009; Zamunar et al. 2019). Our findings confirm that aquatic exercises, when self-paced and with appropriate rest intervals, will not exacerbate fatigue or PEM (Busch et al. 2011; Castro-Sanchez et al. 2012; Goudsmit et al. 2012; Broadbent et al. 2018).

A further very important finding from the current study was the significant decrease in depression after the intervention. Both the total HADS scores (combined depression and anxiety) and the depression component decreased, which is very important given that 40.6% of participants had been diagnosed with clinical depression and were taking anti-depressants, and 28.1% had an anxiety disorder. Earlier studies noted that aquatic exercise improved anxiety/depression, emotional and mental health in fibromyalgia patients (Jentoft et al. 2001; Tomas-Carus et al. 2009; Busch et al. 2011; Bekaryssova et al. 2025), with similar findings for aquatic therapy for MS patients (Castro-Sanchez et al. 2012) and older adults with depression (da Silva et al. 2019). Furthermore, there is growing evidence that moderate-intensity strength training can improve mood, depression and confusion in individuals with FMS (Andrade et al. 2019). Exercise and PA are recommended management strategies for mental health conditions (Schuch et al. 2016; da Silva et al. 2019; Jackson et al. 2022) with substantial evidence that they can improve neurotransmitter and neurohormonal levels (e.g., endorphins, dopamine, serotonin, brain-derived neurotrophic factor, insulin-like growth factor) (Kang et al. 2020; Bekaryssova et al. 2025), cognition (Kang et al. 2020), cerebral blood flow, sleep, oxidative stress (da Silva et al. 2019) and quality of life (Chen et al. 2022). Interestingly, individuals receiving both exercise and anti-depressants showed greater improvements in cognition and autonomic balance than those using medications alone (Neviani et al. 2017; Chen et al. 2022) which suggests an adjunct role of PA with pharmacology for managing depression and anxiety. It is also worth noting that exercise sessions delivered by a qualified exercise professional and in a group setting tend to show a greater beneficial effect on depression (Schuch et al. 2016). This finding is supported by qualitative evidence from ME/CFS patients and other chronic illness groups, where appropriate delivery and group support can improve exercise compliance (Rae and White 2009; Salem et al. 2011; Broadbent et al. 2020; Strassheim et al. 2021).

We found significant post-intervention changes in some of the secondary outcome measures. As lung function was not assessed in our previous pilot study, we cannot compare values from this 6-month trial. Peak expiratory flow (PEF) significantly increased for the INT group although the other pulmonary variables did not change. The most likely explanation for improved PEF is the strengthening of the intercostal muscle and diaphragm in response to immersion and exercising in chest-deep water (Becker 2009), which would increase the ability to breathe out forcefully. Similar findings have been reported with COPD patients undertaking aquatic rehabilitation (de Souto Arauja et al. 2012; McNamara et al. 2013; Felcar et al. 2018). Our exercise program was low-intensity so that it might account for the lack of change in lung volumes, yet the program provided enough stimuli to enhance respiratory muscle strength (Becker 2009). This might also contribute to enhanced exercise tolerance (de Souto Araujo et al. 2012; McNamara et al. 2013; Felcar et al. 2018). Interestingly, the CON group showed a trend to reduced FVC and FEV1, suggesting either mild deterioration in lung function or poorer attempts when performing spirometry during post-intervention testing.

There were no changes in Sit–Reach scores pre-to-post, or of Apley Shoulder Test scores, in either group. The CON group demonstrated more hamstring flexibility post-study compared to the INT group. We suggest caution when interpreting this result because of the large standard deviations in both groups for each of these outcome measures. The INT group completed stretching of all main muscle groups at each aquatic session during 6 months, but this did not result in a significant improvement in shoulder and hamstring flexibility. There may be contributing factors, such as variance in symptoms and pain on the days of testing, and greater variance with the large sample sizes in the RCT.

Full blood counts were conducted pre- and post-intervention to assess if any participants had low concentrations of leukocytes or red blood cells. The INT group did show lower platelet counts post-intervention which may be due to reduced platelet aggregation with regular exercise, a potential cardioprotective benefit in terms of reduced risk of thrombosis (Haynes et al. 2018). Monocyte concentrations had been lower in the INT group compared to CON before the study, with levels increasing post-intervention. Monocytes can exert both anti- and pro-inflammatory effects depending on their phenotype. Ninety percent of monocytes are the classical CD14 +  + phenotype that can migrate to, and reduce, areas of inflammation. Ten percent are CD16 + 14 + cells that can produce pro-inflammatory proteins; this cell population may be elevated in patients with cardiovascular disease, diabetes and rheumatoid arthritis. (Timmerman et al. 2008). Regular physical activity and exercise can decrease the activity of inflammatory monocytes and enhance the function of anti-inflammatory monocytes and macrophages. We could speculate that regular physical activity enhanced the monocyte concentration in our participants which would improve immunosurveillance and reduce risk of inflammatory conditions and infections (Timmerman et al. 2008).

Intervention adherence was 75%. Shorter duration aquatic studies have found higher adherence (e.g., 86.9% and 88%) (Broadbent et al. 2018; Salem et al. 2011), but it was expected that attendance would be lower on average over the much longer time period. Some participants were more symptomatic than others; some participants missed only one or two sessions in the entire 6 months, while others had more frequent absences. Reasons for missing sessions included general tiredness, occasional lower back pain, seasonal illnesses (colds and flu), medical/other appointments that could not be rearranged, no transport on the day, family-related matters and scheduled holidays. It is important to note that none of the participants reported missed aquatic sessions because of intervention-related symptom exacerbation. We suggest that self-paced aquatic exercise can be considered safe as a mode of rehabilitation since no harmful incidents were reported by participants or supervising staff (e.g., slips, falls, injuries, PEM, worsening symptoms). However, we also stress that in the interest of client safety and efficacy of the exercise program, the session should be instructed by an exercise professional (therapist or clinician) who is qualified in rehabilitation exercise for clinical cohorts and who understands the physical and mental limits of the patients. It is clear from qualitative studies that patients respond better to instructors who understand the chronic condition, symptoms and limitations; who communicate and demonstrate the exercises well; and who provide exercises that are manageable for participants (Broadbent et al. 2020; Strassheim et al. 2021).

The physical and psychosocial advantages of aquatic PA include improved cardiovascular, respiratory, strength, balance and quality of life (Fail et al. 2021), reduced pain, fatigue, mental health and cognitive symptoms (da Silva et al. 2019; Zamunar et al. 2019; Jackson et al. 2022) and high levels of enjoyment and social interaction (Rae and White 2009; Castro-Sanchez et al. 2012; Broadbent et al. 2020). Regarding potential disadvantages compared to land-based PA, there may be a higher risk of slips and falls on wet surfaces and there are some contraindications for water immersion, such as open wounds, skin infections, allergies to pool chemicals, fear of water (or being a non-swimmer), and severe cardiac and lung diseases (Becker 2009). Access to rehabilitation pools, travel concerns and cost may be inhibiting factors although some of these issues also apply to accessing land-based exercise facilities. The benefits associated with aquatic PA are still likely to outweigh the physical risks but cost-effectiveness, transport and accessibility may be barriers to participation.

### Study limitations

Although an improvement on the pilot study numbers, the combined sample size of this RCT was not large, and for many outcome measures, there was considerable variance. We suggest that this reflects the range of severity of ME/CFS in the general population. This study was conducted at two sites, one of which is rural and the other on the geographical northern border of metropolitan. Distance and travel can be barriers to participation for some individuals with ME/CFS, affecting the potential sample size. However, our study size was similar to other studies which investigated aquatic exercise for clinical cohorts, and there is support for our findings with many outcome measures. This RCT also replicates improved results from the pilot study (e.g., walk distance, FACIT scores).

## Conclusion

Six months of self-paced, low-moderate aquatic exercise significantly improved fatigue, depression, walk distance, lower limb strength and peak expiratory flow. PEM was not worsened by the intervention, which is extremely important for ME/CFS management. This mode of physical activity may be safe, effective in improving functional capacity, and manageable for individuals with ME/CFS.

## Supplementary Information

Below is the link to the electronic supplementary material.

Supplementary file1 (DOCX 69 KB)

Supplementary file2 (DOCX 19 KB)

## Data Availability

Deidentified data may be supplied upon request to the research team.
