# Supplementary material for: Physical function and psychosocial outcomes after a 6-month self-paced aquatic exercise program for individuals with myalgic encephalomyelitis/chronic fatigue syndrome
Source: Eur J Appl Physiol. 2025 Apr 5;125(9):2515–28. doi: 10.1007/s00421-025-05759-5 (PMC12423189; doi:10.1007/s00421-025-05759-5)
Supplement: Supplementary file 1 — Supplementary file1 (DOCX 69 KB) [file 421_2025_5759_MOESM1_ESM.docx]

Fig 1 Comparison of 6MWT distances for INT and CON participants Pre-Post intervention

Fig 2 Comparison of Sit-to-Stand Test scores for INT and CON participants Pre-Post intervention

Fig 3 Comparison of Left Hand Grip scores for INT and CON participants Pre-Post intervention

Fig 4 Comparison of Right Hand Grip scores for INT and CON participants Pre-Post intervention

Fig 5 Comparison of Sit-Reach Test scores for INT and CON participants Pre-Post intervention

Fig 6 Comparison of Apley’s Shoulder Test (Left) scores for INT and CON participants Pre-Post intervention

Fig 7 Comparison of Apley’s Shoulder Test (Right) scores for INT and CON participants Pre-Post intervention

Fig 8 Comparison of FACIT scores for INT and CON participants Pre-Post intervention

Fig 9 Comparison of Total HADS scores for INT and CON participants Pre-Post intervention

Fig 10 Comparison of HADS Depression scores for INT and CON participants Pre-Post intervention

Fig 11 Comparison of HADS Anxiety scores for INT and CON participants Pre-Post intervention

**Footnotes**

**Supplementary** **Fig 1 – 11** INT group – solid lines; CON group – dash lines

Fig 9-11 – red lines (solid or dash) show participants taking anti-depressant medications
