# Supplementary material for: Physical function and psychosocial outcomes after a 6-month self-paced aquatic exercise program for individuals with myalgic encephalomyelitis/chronic fatigue syndrome
Source: Eur J Appl Physiol. 2025 Apr 5;125(9):2515–28. doi: 10.1007/s00421-025-05759-5 (PMC12423189; doi:10.1007/s00421-025-05759-5)
Supplement: Supplementary file 2 — Supplementary file2 (DOCX 19 KB) [file 421_2025_5759_MOESM2_ESM.docx]

Supplementary Table Participant medications and supplements

| **Medications, vitamins and supplements** | **Frequency INT group (n=17)** | **Frequency CON group (n=15)** | **Percentage of cohort**  **(n=32)** |
| --- | --- | --- | --- |
| Anti-depressants  Anxiety / other MH medications  Neuropathic pain medications  Sleep medications  Thyroxine  Beta-blockers/other BP  Aspirin/other blood thinners  Prolia  Cancer-specific  Ventolin/other pulmonary  Proton pump inhibitors  Statins  Prediabetes / diabetes  Panadol  Ibuprofen/other anti-inflammatories  Adrenal hormone  Naltrexone  Testosterone  Clonazepam (epilepsy)  Vitamins  Probiotics  Minerals (iron, zinc, magnesium, selenium)  Co-enzyme Q10  Fish oil and/or Omega 3  Astragalus  Turmeric  Calcium  Other herbs | 7  4  3  2  2  2  1  1  1  3  2  2  0  3  1  1  2  0  1  8  1  11  2  2  1  1  3  1 | 6  2  3  1  5  2  2  0  1  1  3  1  1  3  1  1  0  1  0  12  0  9  0  1  1  0  2  6 | 40.6%  18.6%  18.6%  9.4%  21.9%  12.5%  9.4%  3.1%  6.3%  12.5%  15.6%  9.4%  3.1%  18.6%  6.3%  9.4%  6.3%  3.1%  3.1%  62.5%  3.1%  62.5%  6.3%  9.4%  6.3%  3.1%  15.6%  21.9% |
